# 

**Published:** 1917-04-21

**Authors:** 


The name of Lieut.-Col. H. L. Battersby, who is in charge of the military hospitals in Nottingham, appears in the list of officers brought to the notice of the Secretary of State for War for valuable services in connection with the war.

### Hospital and Institutional Results.

**Royal Aberdeen Hospital for Sick Children**—"Don't let the Flag of Mercy fall, or doom to death's defeat the children small, whose ceaseless call the hospital must meet." This appealing verse prefaces the fortieth annual report. The hospital buildings proper being temporarily used for war purposes, the in-patient work of the charity has again been carried on at Kepplestone, while the out-patients were looked after by the Royal Infirmary for the first half of the year, when the old out-patient department was reopened in Castle Terrace.

**East End Mothers' Lying-in Home.**—A feature of the report is an account of the work, written by the resident lady superintendent, who says: "Statistics in our work are rather useless because the statistics, to be of any national value, should be taken four years hence, by numbering the babies born in 1916 who have worried through these trying first years."

**St. George's Hospital.**—The ordinary income of the hospital was £30,689 and the total expenditure £48,806, but the year was an unusually good one as regards receipts from legacies, which totalled £34,725, the cash position being that the hospital is enabled to face a deficit this year, should one arise, with a substantial balance in hand. The annual report has been abbreviated for the sake of economy.

**Norfolk and Norwich Hospital.**—The report appears in its customary form. 1,432 patients from the British Expeditionary Force were received during 1916, making a total of 4,650 since the arrival of the first convoy. The total income was £30,766 and the total expenditure £31,898. There was an increase in cost of £1,917 under the heading of provisions as compared with the previous year.

**Ancoats Hospital, Manchester.** — Three of the honorary staff, who had left the hospital for military duties, have been able to renew their work at the hospital. In-patients, out-patients, and casualties all decreased in numbers during 1916. There was a deficiency in the accounts of £1,170, and the debt to the bank on December 31 was £4,135.

### Matrons' and Sisters' Appointments.

Birmingham and Midland Counties Sanatorium.—Miss V. Cooper, whose appointment as matron we announced in our last issue, was trained at Derbyshire Royal Infirmary, and held subsequently the following appointments: Sister and matron's assistant at the Walsall General Hospital, military sister in France and Belgium during the first year of the war, and home sister, housekeeper, and assistant matron at the Hospital for Sick Children, Great Ormond Street.

City of Westminster Union Infirmary, Fulham Road.—Miss M. R. M. Boniface has been appointed night superintendent. She was trained at St. Marylebone Infirmary, was then staff nurse at Charing Cross Hospital, night superintendent and labour ward sister at the East End Lying-in Home, London, and sister at a private hospital in Melbourne, and at St. Margaret's Hospital, Sydney. Miss Boniface was also bush nurse in Gippsland, Victoria, and has done private nursing in England and Australia and military nursing in England and France.

Birkenhead Borough Hospital.—Miss E. M. Edwards has been appointed night sister. She was trained at Erdington Infirmary, Birmingham, and has since been sister at Steyning Union Infirmary.

Chesterfield and North Derbyshire Hospital.—Miss L. Browne has been appointed night sister. She was trained at the Bolton Infirmary and the Monsall Fever Hospital, and besides doing private nursing has been sister at Merthyr General Hospital, at Bolton Infirmary, and at Rochdale Infirmary.

Edinburgh Women and Children's Hospital.—Miss Ada L. Norman has been appointed night sister. She was trained at the Royal Infirmary, Huddersfield, has been staff nurse at the General Hospital, Ramsgate, and sister at the Alexandra Hospital, Montreal, and at the Children's Hospital, Bradford. She has also done private nursing.

Jessop Hospital for Women, Sheffield.—Miss Margaret Barrow has been appointed theatre sister. She was trained at Clayton Hospital, Wakefield, where she later held the post of staff nurse. She has been sister at the General Hospital, Kettering, and at York County Hospital, and temporary assistant matron at Lincoln County Hospital.

Royal Aberdeen Hospital for Sick Children.—Miss Clara Margaret Woodward has been appointed sister. She was trained at Rotherham Hospital and Dispensary, and has been staff nurse at the Hospital for Epilepsy and Paralysis, Maida Vale.

Reigate and Redhill General Hospital.—Miss Winifred Leigh and Miss Gertrude Marshall have been appointed sisters. Miss Leigh was trained at Reigate Hospital; Miss Marshall at Leicester Royal Infirmary, where she was later holiday sister, and undertook sister's duties.

St. Chad's Hospital, Birmingham.—Miss Mary Edwards has been appointed sister. She was trained at Queen's Hospital, Birmingham, where she was later sister, and has since been sister at Great Barr Hall, Birmingham.

St. Bartholomew's Hospital, Rochester.—Miss B. Henman has been appointed theatre sister. She was trained at St. Bartholomew's Hospital, London, E.C.

Royal Buckinghamshire Hospital, Aylesbury.—Miss Pollie Wood has been appointed sister of the men's ward. She was trained at Derbyshire Royal Infirmary.

Military Hospital, Wellington.—Miss Adèle Tudor Dodd has been appointed sister. She was trained at Bristol General Hospital and also at the City Hospital, Bristol. She has been sister of the military ward at the Royal Liverpool Hospital, Heswall.

**NOTE.—Particulars of Appointments for insertion in this column will be welcomed.**

### Passing Events and Topics.

### The Royal School for Deaf and Dumb Children, Margate.

The special Easter appeal of the Royal School for Deaf and Dumb Children takes the form of an attractive yet inexpensive booklet illustrating the educational and industrial training at the school.

### Hospital Saturday Fund.

Sir T. Vezey Strong, Bart., presided at the recent annual meeting of the Hospital Saturday Fund. The report of the Council showed that the receipts for 1916 reached £56,610, as against £40,750 in the previous year.

### Marlborough School of Mothercraft.

The Right Hon. W. F. Massey, Premier of New Zealand, will open the Marlborough School of Mothercraft, 29-31 Trebovir Road Earl's Court, on Tuesday, April 24. The chair will be taken at 3.30 by the Mayoress of Kensington.

### Queen Mary's Naval Hospital, Southend.

Since the opening of Queen Mary's Royal Naval Hospital, Southend, 169 Belgian soldiers and 3,382 British soldiers and 744 sailors have been treated. The accommodation, which has been increased to 350 beds, will now be used exclusively for sailors.

### Zenana Bible and Medical Mission.

The annual meeting of the Zenana Bible and Medical Mission will be held at Sion College on Tuesday, April 24, at 3 p.m. The speakers will include Sir Andrew Fraser, K.C.S.I. (in the chair), Dr. Ethel Landon, of the Canadian Hospital, Nasik, and Miss Leetch, of Allahabad.

### An Historic School.

The Annunziata School at Florence, where the Queen of the Belgians has just placed her daughter, Princess Marie José, is a place of historic interest. It was originally a villa belonging to the Medici family, but later came into the possession of the royal House of Savoy. While on a visit as an infant to the then King and Queen of Sardinia, King Victor Emanuel, the first ruler of United Italy and the grandfather of the present king, was nearly burnt to death. By some accident the mosquito curtains of his cot caught fire, and he was saved only by the devotion of his nurse, who snatched him up and extinguished the flames. The nurse herself was so severely burnt that she died within three weeks, but the hope of Italy was saved.

### The Book Market.

LIST OF NEW BOOKS RECEIVED FROM THE FOLLOWING PUBLISHERS:—

Cassell and Co.: "A Handbook of Midwifery." By Comyns Berkeley, M.A., M.D. 6s. net.

"Notes on Military Orthopædics." By Colonel Robert Jones, C.B. 2s. 6d. net.

Longmans, Green and Co.: "Common Diseases of the Male Urethra." Frank Kidd, M.B. 5s. net.

Cambridge University Press: "Science and the Nation." Edited by A. C. Seward, F.R.S. 5s. net.

### Papers, Pamphlets, etc.

Transactions of the Incorporated Sanitary Association of Scotland. 1916.

Report of the Director-General of Public Health, New South Wales, 1914.

### Manager's Notices.

**All letters on business matters must be addressed to The Manager, "The Hospital," 28 and 29 Southampton Street, London, W.C. 2.**

**Subscriptions**, which may commence at any time, are payable in advance. Cheques and Post-Office Orders should be crossed London County and Westminster Bank, Covent Garden Branch, and made payable to the Manager of The Hospital.

Rates of Subscription (payable in advance).

| | | |
|---|---|---|
| UNITED KINGDOM. | | |
| Three Months | (including Postage) | 2s. 0d. |
| Six Months | do. | 4s. 0d. |
| Twelve Months | do. | 6s. 6d. |
| FOREIGN AND COLONIAL. | | |
| Three Months | (including Postage) | 2s. 6d. |
| Six Months | do. | 5s. 0d. |
| Twelve Months | do. | 8s. 8d. |

